# Observation of Cytotoxicity of Phosphonium Derivatives Is Explained: Metabolism Inhibition and Adhesion Alteration

**DOI:** 10.3390/antibiotics12040720

**Published:** 2023-04-06

**Authors:** Pavel A. Nazarov, Svetlana A. Khrulnova, Andrew G. Kessenikh, Uliana S. Novoyatlova, Svetlana B. Kuznetsova, Sergey V. Bazhenov, Alexandra I. Sorochkina, Marina V. Karakozova, Ilya V. Manukhov

**Affiliations:** 1 Moscow Institute of Physics and Technology,141700 Dolgoprudny, Russiamanukhovi@mail.ru (I.V.M.); 2 Belozersky Institute of Physico-Chemical Biology, Lomonosov Moscow State University, 119992 Moscow, Russia; 3 National Research Center for Hematology, 117198 Moscow, Russia; 4 Laboratory for Microbiology, BIOTECH University, 125080 Moscow, Russia; 5 Center of Life Sciences, Skolkovo Institute of Science and Technology, 121205 Moscow, Russia

**Keywords:** MTT test, cytotoxicity, phosphonium, cytostatic, bioluminescence, biosensor, antibiotic

## Abstract

The search for new antibiotics, substances that kill prokaryotic cells and do not kill eukaryotic cells, is an urgent need for modern medicine. Among the most promising are derivatives of triphenylphosphonium, which can protect the infected organs of mammals and heal damaged cells as mitochondria-targeted antioxidants. In addition to the antioxidant action, triphenylphosphonium derivatives exhibit antibacterial activity. It has recently been reported that triphenylphosphonium derivatives cause either cytotoxic effects or inhibition of cellular metabolism at submicromolar concentrations. In this work, we analyzed the MTT data using microscopy and compared them with data on changes in the luminescence of bacteria. We have shown that, at submicromolar concentrations, only metabolism is inhibited, while an increase in alkyltriphenylphosphonium (CnTPP) concentration leads to adhesion alteration. Thus, our data on eukaryotic and prokaryotic cells confirm a decrease in the metabolic activity of cells by CnTPPs but do not confirm a cytocidal effect of TPPs at submicromolar concentrations. This allows us to consider CnTPP as a non-toxic antibacterial drug at low concentrations and a relatively safe vector for delivering other antibacterial substances into bacterial cells.

## 1. Introduction

The search for antimicrobial drugs is a pressing need for modern science. It is not difficult to find an abiotic, a substance that kills all living things, while the search for antibiotics, substances that kill bacteria and do not kill eukaryotic cells, is a difficult task. Among the most promising antibacterial agents are mitochondria-targeted antioxidants, which can effectively kill bacterial cells, cure damaged host cells, and reduce hypercytokinemia [1,2,3].

Mitochondria are the cellular powerhouses that generate cellular energy in the form of adenosine triphosphate, and the mitochondrial matrix is negatively charged. Healthy mitochondria have a membrane potential of approximately −180 mV and, due to the negative membrane potential of the inner membrane of mitochondria, positively charged compounds must accumulate in the mitochondrial matrix against their concentration gradient, which is the basis for the use of positively charged mitochondria-targeted antioxidants (MTA). Various lipophilic, positively charged cations accumulate inside mitochondria according to the Nernst equation. Skulachev’s group [4,5,6] proposed to use triphenylphosphonium derivatives as mitochondria-targeted substances, with TPP-conjugated molecules serving as a “locomotive” for mitochondria targeting. Two decades later, Murphy and co-workers [7,8,9] reinvented this approach to the delivery of antioxidant moiety to mitochondria, and many molecules based on triphenylphosphonium derivatives have been created [10]. Despite the relative similarity of mitochondria and bacteria, it has long been thought that mitochondria-targeted antioxidants such as SkQ1 lack antibacterial properties [11,12]. However, we have shown that alkyltriphenylphosphonium cations (CnTPP) and SkQ1 have a strong antibacterial effect on Gram-positive and Gram-negative bacteria [1,13]. Moreover, SkQ1 has a bactericidal effect, which makes it promising for use in clinical practice. Even an intensive study of triphenylphosphonium derivatives did not allow us to fully understand the mechanism of their action. Although it is known that they reduce the membrane potential of mitochondria and bacteria, the mechanism of this decrease remains unknown. The most known probable mechanism is their protonophore-like action due to free fatty acids [14], which, apparently, should be universal for mitochondria, prokaryotes, and eukaryotes.

The mechanisms of resistance to triphenylphosphonium derivatives are different for prokaryotes and eukaryotes. At the moment, the mechanism of resistance of Gram-positive bacteria, if any, is not apparent. The mechanism of resistance of some Gram-negative bacteria depends on the presence of the MDR pump AcrAB-TolC [1,2], or similar functional paralogs [15]. Although, according to the theory, a greater accumulation of triphenylphosphonium derivatives should be observed in the mitochondria of eukaryotic cells [16], the cytotoxic effect for eukaryotic cells was observed only in the micromolar range. For example, C_10_TPP began to affect Jurkat human T lymphocyte cell viability at a concentration of 1–2 µM [17]; mitoFluo (C_10_TPP derivative with fluorescein) at a concentration of 1 μM and 2 μM on Rko cells and on L929 fibrosarcoma cells, respectively [18,19]; and for SkQ1 (C_10_TPP derivative with plastoquinone) on HeLa cells, toxicity began to appear at 2 μM, and 20 μM caused only 50% toxicity [1]. This is also confirmed by cells of *Saccharomyces cerevisiae* yeast having a MIC of approximately 30 μM [20]. Recently, a work was published in which the authors reported on cytotoxicity of triphenylphosphonium derivatives (CnTPP, where n = 8–18) in relation to human embryonic 293T cells at submicromolar concentrations [21].

Since triphenylphosphonium derivatives are becoming more and more popular vectors for delivering antibacterial substances to cells [22,23], finding out whether the cytocidal effect in submicromolar concentrations can seriously affect the development of newer antibacterial drugs is important. Cytotoxicity plays an important role in the validation of substances as promising antibacterial agents; therefore, not understanding the reasons leading to apparent cytotoxicity can seriously hinder the development of new antibacterial agents that modern healthcare needs so much. In this work, we show that the observed cytotoxicity is the result of metabolic inhibition and adhesion alteration; thus, at submicromolar concentrations, it is only imaginary.

## 2. Results

### 2.1. C_12_TPP Inhibits Bacterial Metabolism

In our former works, we analyzed the antibacterial action of CnTPP (where n = 4–14) against bacteria *Escherichia coli* and *Bacillus subtilis* [13,23]. It was shown that with increasing length of the alkyl fragment, antibacterial activity increases, reaching its maximum at n = 12, and then decreases with increasing length of the alkyl fragment. Thus, for our experiments, we chose C_12_TPP, which had the maximum antibacterial activity.

Although it has previously been shown that triphenylphosphonium derivatives decrease ROS (reactive oxygen species) production due to the accumulation in mitochondria [24], a direct decrease in metabolic activity has not been shown, although conclusions about a possible effect on the inhibition of metabolic processes could be expected [14]. To test this, we decided to compare the reduction in luminescence caused by the action of C_12_TPP and the survival of bacteria, expressed in CFU. The difference in the results will allow us to confirm the decrease in metabolism, while the absence of a difference will show that the level of luminescence falls due to cytotoxicity.

#### 2.1.1. C_12_TPP Causes a Drop of Luminescence at Submicromolar Concentrations

To confirm that C_12_TPP inhibits metabolism, we have chosen luminous bacteria. The following strains were used: naturally luminescent Gram-negative bacteria *Vibrio aquamarinus* VNB 15 and genetically modified strains of *E. coli* and *B. subtilis* carrying bacterial *lux* operon of *Photorhabdus luminescens* [25,26,27]. For Gram-positive *B. subtilis*, luminescence began to decrease at submicromolar concentrations of C_12_TPP, while Gram-negative bacteria *E. coli* and *V. aquamarinus* appeared to be more resistant, with a decrease in their luminescence observed at concentrations of 1–2 μM and above (Table 1). These values were at least an order of magnitude lower than the minimum inhibitory concentrations (MIC) for both *B. subtilis* and *E. coli*. Thus, for a correct assessment of the contribution of a decrease in the membrane potential, it is necessary to measure the reduction in the survival rate under equivalent conditions.

#### 2.1.2. C_12_TPP Causes Reduction of Survival Rate

For each concentration that we used, we measured the threshold at which a bactericidal effect was observed. As expected, for Gram-positive *B. subtilis*, it turned out to be the minimum bactericidal concentration (MBC) in the range of 1–2 μM, which we have demonstrated earlier for other Gram-positive bacteria [1,13,28]. Thus, the concentration triggering the reduction of luminescence is an order of magnitude lower than the concentration at which the bactericidal action begins.

For Gram-negative *E. coli* and *V. aquamarinus*, we see a similar situation. MBC is in the range of tens of micromoles, which is also an order of magnitude higher than the luminescence reduction concentration.

**Figure 1:**
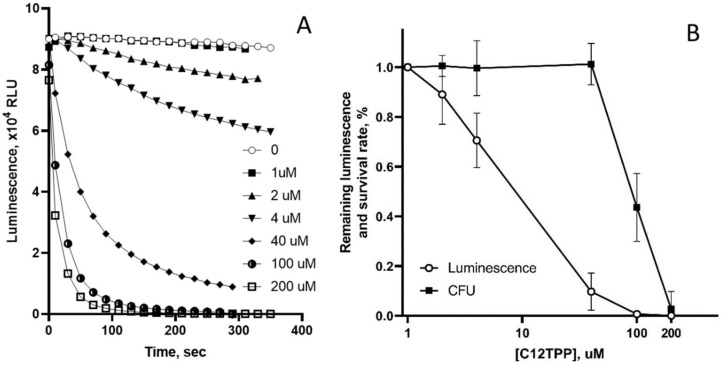
(**A**) Time dependence of the bioluminescence intensity of lux-biosensors *E. coli* MG1655 (pXen7) carrying a *lux* operon of *P. luminescens* in the presence of C_12_TPP at different concentrations. (**B**) Comparison of luminescence and survival rate of *E. coli* MG1655 (pXen7) cells after 15 min incubation with C_12_TPP at different concentrations.

To obtain a more complete picture, we titrated C_12_TPP by measuring luminescence and CFU for *E. coli* (Figure 1). Surprisingly, at a concentration of 40 µM, we observed an almost 90% decrease in the level of luminescence, but there was no reduction in survival (Figure 1B). Thus, the decrease in luminescence is not the result of cell death, as the cells remain viable.

### 2.2. C_12_TPP Inhibits Metabolism of Mammalian Cells

Unlike that of bacteria, the viability of a mammalian cell culture is often determined by a colorimetric test to assess the metabolic activity of cells (MTT, MTS, XTT, and WST). Since such a test does not directly evaluate viability, unlike CFU, it has a number of limitations hindering its usage; the results can be erroneous due to: (1) the depletion of essential nutrients, (2) the uneven cell density, (3) the uncoupling of oxidative phosphorylation, (4) the adhesion alteration, etc. The simplest method for assessing cytotoxicity is direct microscopy.

#### 2.2.1. CnTPP Does Not Show a Decrease in Viability at Submicromolar Concentrations

We decided to test the cytotoxicity of these derivatives against human embryonic 293T cells by means of microscopy control. When adding concentrations exceeding the GI50, GI80, and GI90 used in work [21] for these substances, cytotoxicity was not observed (Table 2). Cells retain ~100% confluence at the concentrations studied in [21], which indicates the absence of cell death and, consequently, cytocidal activity. This allows us to assert that the effect observed by the authors [21] is not cytotoxic but cytostatic with possible metabolism inhibition. Clearly, even with GI90, as defined by the term itself, a 10% increase in cell population is observed compared to the control, meaning that the conditions could not be considered cytotoxic even formally. At the same time, the cells have a visually unchanged shape and do not differ in any way from those not treated with CnTPP. Thus, we can conclude that the addition of CnTPP does not lead to apparent cytotoxicity and its effect is probably the result of a decrease in the activity of NADPH-dependent cellular oxidoreductases, which does not necessarily lead to cell death.

For further research, as in the case of bacteria [13], we chose the most “toxic” CnTPP molecule, C_12_TPP.

#### 2.2.2. A Decrease in Viability at Submicromolar Concentrations of C_12_TPP Is Shown by MTT, but Not by Microscopy

**Figure 2:**
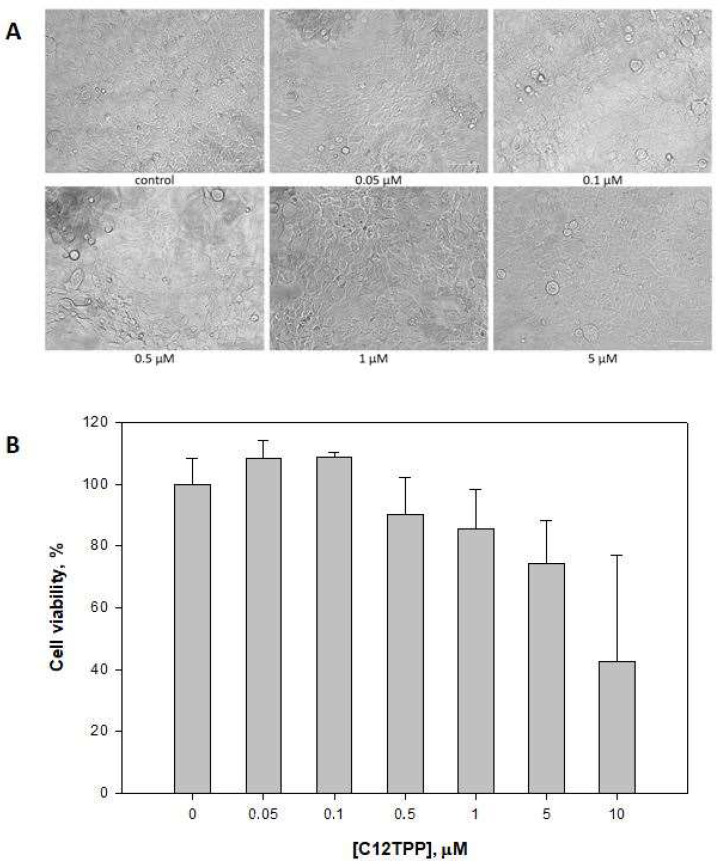
Microscopy of 293T cells in a 96-well plate prior to MTT test (**A**). In each well of 96-well plate, monolayers remain confluent over the entire concentration range from 0.05 µM to 5 µM; viability of 293T cells in accordance with MTT test (**B**).

We continued to study the cytotoxic effect of C_12_TPP in the concentration range from 0.05 μM to 10 μM. We performed a standard cytotoxic test and microscoped each well before MTT assay. For this, we added the appropriate concentration of commercially available C_12_TPP to 293T cells in DMEM (with 2 mM L-glutamine and 10% fetal bovine serum) and cultivated them at 37 °C in 5% CO_2_/95% air for 24 h. The results of our experiment are shown in Figure 2.

No significant difference in monolayer confluence, and therefore absence of cytotoxicity, was observed up to the concentration of 5 μM (Figure 2A). However, at concentrations above 1 μM, a slight change in cell morphology was observed. It is known that the cytotoxic effect can be detected by changes in cell morphology [24]; therefore, the observed change in morphology at micromolar concentrations correlates with previously obtained data on the cytotoxicity of CnTPPs [1,17,18,19,20].

It is noteworthy that the cytotoxicity measured by the MTT test did not correlate with confluence estimation obtained via microscopy. At a concentration above 0.1 μM, an increased variability of the results was observed, which did not correlate with our previously obtained microscopic data on the same samples. We hypothesize that this could be due to metabolism inhibition or a change in cell adhesion caused by the incorporation of charged triphenylphosphonium molecules into the cell membrane.

#### 2.2.3. C_12_TPP Is Indicative of Impaired Cell Adhesion

**Figure 3:**
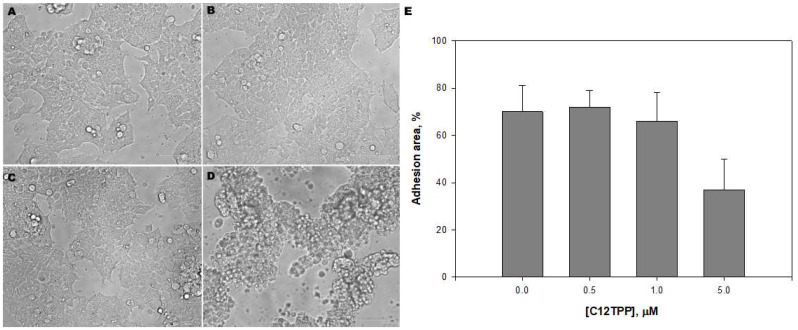
Microscopy of 293T cells in a 96-well plate after C_12_TPP preincubation: (**A**) control without C_12_TPP, (**B**) 0.1 μM, (**C**) 1 μM, (**D**) 5 μM, and (**E**) determination of the adhesion area. The adhesion area was analyzed and measured using ImageJ software. The clumpy cell phenotype increased with increasing TPP concentration and reached a maximum at 5 µM.

To find out if triphenylphosphonium derivatives affect cell adhesion, we preincubated the cells with C_12_TPP and placed them in a 96-well plate to form a cell monolayer. Cells attached to the surface and formed a monolayer at submicromolar and micromolar concentrations of C_12_TPP (see Figure 3). At the same time, with increasing concentration, the usual phenotype changes to clumpy and reaches a maximum already at 5 μM C_12_TPP. Concurrently, the concentrations used by us do not cause serious effects when added to the cell monolayer, which excludes the presence of a toxic effect as a reason for the formation of a clumpy phenotype. Thus, the clumpy phenotype is not a consequence of a high concentration of C_12_TPP. The clumpy phenotype is due to the accessibility of the cell membrane to C_12_TPP and the impossibility of attaching to the surface when C_12_TPP is inserted into the membrane.. At the same time, cells in the clumpy phenotype can bind to the surface, but these bonds are very weak and are destroyed at the slightest impact. Concurrently, the cells on the first day demonstrated the ability to slightly grow. Slow growth continued until the mass death of cells that could not adhere to the surface (up to 80%, according to rough estimates).

Thus, the changes detected by the MTT test for CnTPP at submicromolar concentrations, as in the case of bacterial luminescence, are only a reflection of a decrease in cell metabolism. At concentrations above 1 μM, apparently, some toxic effect begins to appear; however, cell death is not observed even at 5 μM. At a concentration of 10 μM, a large decrease in confluence is observed; however, in this case it is difficult to say whether cell death occurs or only adhesion decreases.

## 3. Discussion

### 3.1. Metabolism Inhibition Is a Factor Affecting the MTT Test Results and Bioluminescence

The MTT assay is a colorimetric assay that measures the reduction of yellow MTT (3-(4,5-dimethylthiazol-2-yl)-2,5-diphenyltetrazolium bromide) to blue formazan. It was the first rapid assay developed for cell viability high screening in a 96-well format [29]. Formazan is produced by mitochondrial dehydrogenases, predominantly mitochondrial succinate dehydrogenase [30], and when cells die, they lose their ability to convert MTT into formazan. Thus, the reduction of tetrazolium dye depends on NADPH-dependent oxidoreductase enzymes and hence on cellular levels of NADPH/NADH.

Bioluminescence is the phenomenon of light emission that results from an enzyme-catalyzed oxidation reaction in living organisms. The core genes, *luxCDABEG*, code for all enzymes involved in the complex machinery enabling bioluminescence. The heterodimeric luciferase LuxAB catalyzes the monooxygenation of long-chain aliphatic aldehydes to the corresponding acids utilizing reduced FMN and molecular oxygen, and the energy released as a photon with a wavelength of about 490 nm [31]. The *lux* operon of *P. luminescens* does not contain the *luxG* gene encoding a NAD(P)H-dependent flavin reductase, which is necessary for the reduction of FMN in luciferase, but *E. coli* cells have their own NAD(P)H-flavin reductase encoded by the *fre* gene. Also NADPH is a source of energy for the acyl protein reductase LuxC, which being complexed with LuxDE is responsible for aliphatic aldehyde synthesis. Therefore, bioluminescence also depends on the level of intracellular NADPH/NADH.

It is known that inhibition of complex I of the respiratory chain decreased the rate of formazan formation, while inhibition of complex IV increased it. At the same time, inhibition of complex III and ATP-synthase only marginally affected the rate of formazan formation [32]. Thus, inhibition of mitochondrial complex I would lead to a drop in the level of NADPH/NADH and tetrazoline reduction. Inhibition of complex I was observed earlier in [33], therefore a decrease in the level of NADPH/NADH would be expected in this case as well. Thus, when measuring cell viability by the MTT method, a false positive decrease is observed, which only corresponds to an inhibition of the work of the mitochondrial complex I. An alternative explanation for the reduced NAD(P)H effect is that CnTPPs may act as mild uncouplers [34,35]. As a result, the membrane potential decreases, which, in turn, can accelerate the work of complex IV and, as a result, reduce the NADH pool. Mild uncoupling can be caused by mechanisms inherent in the mitochondria themselves, for example, by uncoupling by endogenous fatty acids or by the functioning of proteins of the UCP family [36]. In addition, although mild dissociation has its pros and cons, the use of such substances finds its application in medical practice [37,38].

### 3.2. Adhesion Alteration Is a Factor Affecting the MTT Test Results

**Figure 4:**
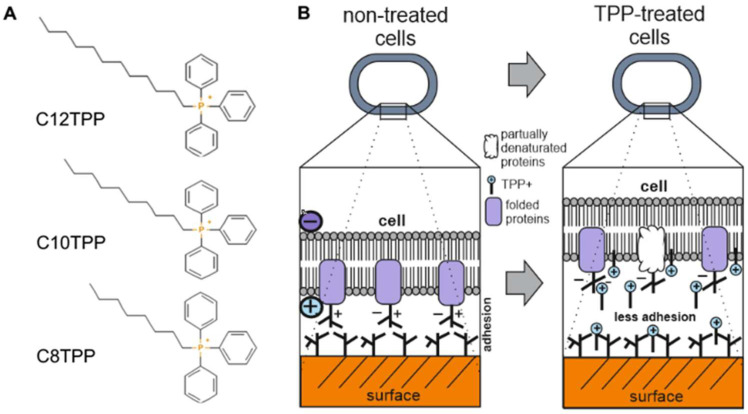
Chemical structures of CnTPP (**A**); hypothesis of cell adhesion alteration (**B**). We assume a decrease in adhesion due to the drop in potential on the membrane and the incorporation of positively charged CnTPP molecules into the membrane of 293T cells.

Adhesion alteration may be an additional factor affecting the MTT test results, because CnTPP is a cationic substance and will be distributed mainly into the cytoplasmic membrane due to (a) its hydrophobic alkyl chain and (b) the positive charge of the TPP head. Embedding in the membrane, CnTPP not only reduces the potential on it, but also leads to a change in the charge of the membrane as a result of its localization in it. In this case, both partial denaturation of protein molecules and disassembly of multisubunit complexes integrated due to electrical and hydrophobic forces can occur. This causes changes in the adhesive properties of the cell membrane, which can lead to the rupture of cell-surface bonds (Figure 4B). Thus, slight perturbations during the MTT test can cause cell dissociation from the surface and lead to measurement errors.

We recently predicted that due to the change in cell adhesion upon the addition of triphenylphosphonium derivatives, an antibiofilm action is expected along with an antibacterial action [39]. Our hypothesis was independently confirmed [28,40], so we can argue that this effect may be responsible for adhesion alteration for 293T cells.

**Figure 5:**
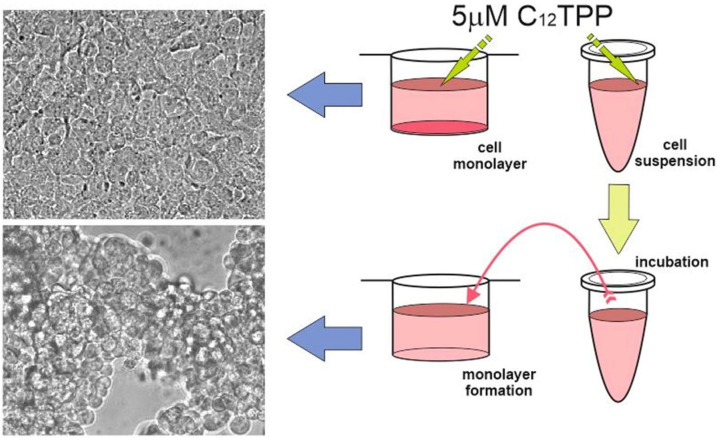
Qualitative experiment confirming adhesion alteration. The same concentration of C_12_TPP was added to the same number of cells in the monolayer and suspension, and after 24 h of incubation, microscopy was performed in a 96-well plate.

We observe a similar result in the case of the experiment with the formation of a monolayer. The cells in the monolayer were insensitive to the action of 5 µM C_12_TPP, while the same cells in suspension were sensitive (see Figure 5). Thus, if there are not enough cells to form an extended monolayer or they do not form monolayers, such as blood cells, then they are sensitive to the action of C_12_TPP. Cells that form monolayers or are capable of forming or incorporating into tissues are not sensitive to micromolar concentrations and only high concentrations of C_12_TPP can affect them. At the same time, it should be noted that cells in the clumpy phenotype are very sensitive to even a small mechanical impact. Apparently, even in the case of a monolayer, cells will be sensitive to various mechanical influences, for example, pouring and adding solutions and media. Since 293T cells are adhesive, and when performing the MTT test, part of the living cells could be removed during measurements and not taken into account when measuring cytotoxicity.

### 3.3. Detergent/Surfactant Effect Does Not Contribute to Bactericidal Action and Toxicity

Although it is clear that the structure of CnTPP molecules is very similar to that of classical surfactants, the main issue is the mechanism of action by which an antibacterial or cytotoxic effect is initiated. It is obvious that at high concentrations (tens of micromoles), a detergent effect can take place. Bacterial *E. coli* cells, yeast *S. cerevisiae*, and human HeLa cells survive at concentrations greater than 20 μM. Thus, at concentrations above 50 μM, it appears that the detergent effect may seriously affect cell survival. At concentrations below 20 µM, the net detergent effect does not seem to play a significant role.

**Figure 6:**
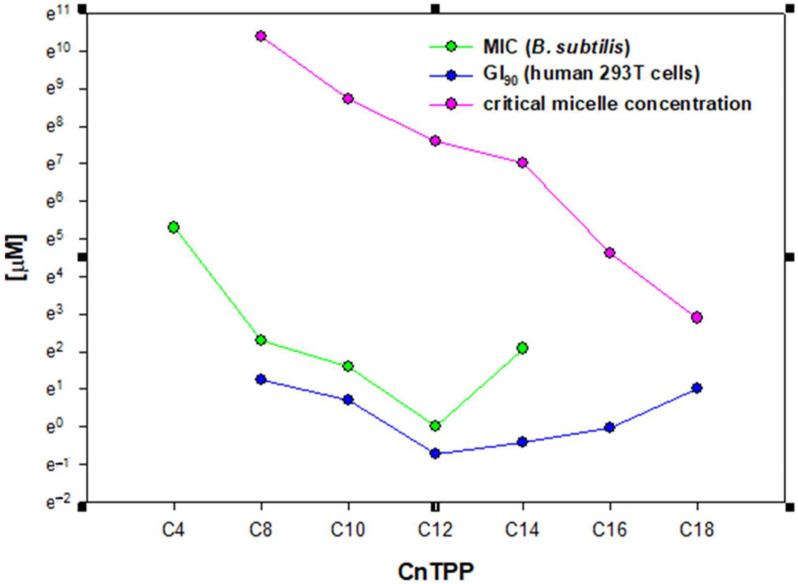
Illustration of the independence of toxic and bactericidal action from the critical micelle concentration (CMC). CMC and toxicity (GI90) are given according to [21], while bactericidal activity is given according to [13,23].

If we look at the critical micelle concentrations (CMC) of CnTPP [21], it is noteworthy that they are 3–4 orders of magnitude higher than GI90 for 293T cells [21] and MIC for *B. subtilis* bacteria [13,23] (Figure 6). As the length of the alkyl chain decreases, the toxicity increases up to C_12_TPP, and then it drops sharply, despite the fact that CMC decreases by 2–3 orders of magnitude. This suggests that CMC is not the cause of the toxicity, which must be caused by another reason.

The protonophoric action itself causes a decrease in cell metabolism due to the formation of a futile cycle (protonophoric cycle) that comes with energy expenditure. However, the incorporation of CnTPP into the mitochondrial or bacterial membrane can apparently lead to disruption of the respiratory chain enzymes. For example, the structure of the c-ring F_1_F_O_ ATP synthase contains additional electron density, which can be occupied by isoprenoid quinones (e.g., coenzyme Q in mitochondria and plastoquinone in chloroplasts) [41]. Thus, TPP derivatives can compete with isoprenoid quinones and interfere with ATP synthase. On the other hand, it is known that the longer-chain CnTPP increased proton leak, decreased maximal respiration and induced an increase in the extracellular acidification rate (ECAR), which is usually interpreted as the production of lactate in glycolysis [33].

The protonophoric action itself causes a decrease in cell metabolism due to the formation of a futile cycle (protonophoric cycle) that comes with energy expenditure. However, the incorporation of CnTPP into the mitochondrial or bacterial membrane can apparently lead to disruption of the respiratory chain enzymes. For example, the structure of the c-ring F_1_F_O_ ATP synthase contains additional electron density, which can be occupied by isoprenoid quinones (e.g., coenzyme Q in mitochondria and plastoquinone in chloroplasts) [41]. Thus, TPP derivatives can compete with isoprenoid quinones and interfere with ATP synthase. On the other hand, it is known that the longer-chain CnTPP increased proton leak, decreased maximal respiration, and induced an increase in the extracellular acidification rate (ECAR), which is usually interpreted as the production of lactate in glycolysis [33].

Since the method measures the activity of mitochondrial enzymes, the signal depends on the level of cellular metabolism [42], cell confluency [43], and also on the depletion of essential nutrients such as glucose or pH alteration [44]. Moreover, as mitochondria-targeted uncouplers of respiratory chain, alkyltriphenylphosphonium cations may alter the redox equilibrium of a cell by changing the levels of reactive oxygen species (ROS) produced during incubation. Although the exact mechanism of respiratory chain inhibition can only be speculated, it can be assumed that CnTPP, penetrating into the mitochondrial and bacterial membranes of living cells, non-specifically affects respiratory chain enzymes and causes a decrease in metabolic activity.

It should be noted that when SkQ1 (a C_10_TPP derivative) is added to mice at concentrations comparable to those bactericidal for Gram-positive bacteria (0.7–1 µmol/ (day·kg body weight)), improvements of health-span and lifespan are observed [45]. This may indicate that the use of CnTPP as a delivery vector or as an antibiotic does not lead to lethal consequences for mammals.

## 4. Materials and Methods

### 4.1. General Comments

Microscopy studies were performed with FLoid Cell Imaging Station (Thermo Fisher Scientific, Waltham, MA, USA). Optical density was measured using a Synergy H4 Hybrid Microplate Reader (BioTek Instruments, Winooski, VT, USA).

### 4.2. Materials

C_12_TPP was purchased from Alfa Aesar (Karlsruhe, Germany). C_8_TPP and C_10_TPP were synthesized previously in accordance with [17]. CnTPP was dissolved in ethanol, while the final concentration of ethanol in any reaction media did not exceed 1%. The addition of ethanol up to 5% does not affect the antibacterial activity and growth of mammalian cells in the presence of CnTPP [1,13]. Components of bacterial media were purchased from Becton Dickinson (Franklin Lakes, NJ, USA) with the following exceptions: Mueller-Hinton medium was purchased from HiMedia Laboratories (Mumbai, India) and agar was purchased from Helicon (Moscow, Russia). Components of mammalian cell culture media were purchased from PanEco LLC (Moscow, Russia). Other reagents were from Sigma-Aldrich (St. Louis, MO, USA).

### 4.3. Bacterial Strains

*B. subtilis* strain 168 trpC2, *E. coli* strain K-12 MG1655 (F-ilvG rfb-50 rph-1), and *V. aquamarinus* strain VNB 15T (B-11245T = DSM 26054T) were obtained from the National Bioresource Center for All-Russian collection of industrial microorganisms (VKPM).

The *E. coli* strain MG1655 transformed by the plasmid pXen7 (plasmid with the inserted *lux* operon of *P. luminescens* under control of its own promoter [25]), *B. subtilis* 168 transformed by the plasmid pNKalkA [46], and *V. aquamarinus* VNB 15T [47] were used as non-specific whole-cell biosensors [48].

Luminescence of the described strains decreases after the addition of a toxic agent due to cell death, metabolic imbalance of the reduced equivalents in the cell, or direct luciferase inhibition.

### 4.4. Culturing of Bacteria

For experiments with bioluminescence measurement, bacterial strains of *E. coli* and *B. subtilis* were grown at 37 °C were grown for 18–24 h on LB agar (Sigma, USA) and then transferred to LB broth and grown until the early exponential growth phase (OD600 = 0.3–0.4 units). If necessary, the medium was supplemented with antibiotics: 100 μg/mL ampicillin or 10 μg/mL chloramphenicol.

*V. aquamarinus* VNB-15 cells were grown at 28 °C on SWT medium (%, wt/vol): tryptone, 0.5; yeast extract, 0.25; sea salt, 1.5; and glycerol, 0.3. Solid medium was prepared using 1.5% agar.

### 4.5. Luminescence Assay

The intensities of bioluminescence (in RLU, relative light units) were measured at room temperature using Biotox-7BM (BioPhysTech, Russia). The bacterial culture (190 μL) was added to the tube and background luminescence was measured. We then added 10 μL of C_12_TPP solution to a final concentration from 0.1 to 200 μM. Stock C_12_TPP solutions were diluted by water to make a concentration of ethanol less 20%; thus, a final concentration of ethanol in cell cultures did not exceed 1%. At this concentration, it does not affect cell luminescence. The intensity of bioluminescence was measured every second for 15 min and expressed in arbitrary units of luminescence. After the measurements, approximately 30 min after C_12_TPP addition, aliquots were used to count CFU.

### 4.6. Mammalian 293T Cell Culture

Then, 293T cells from a human cell line were cultured in Dulbecco’s modified Eagle’s medium (DMEM) supplemented with 2 mM L-glutamine and 10% fetal calf serum, streptomycin (100 U/mL), and penicillin (100 U/mL). Cells were prepared at 100 µL/well in a 96-well plate and cultivated at 37 °C in 5% CO_2_/95% air for 24 h.

### 4.7. 293T Cell Confluency Percentage Estimation

293T cells were grown in flasks for a few days, treated with trypsin (0.25%), harvested, and resuspended in fresh medium to obtain 75–90% confluence on a 96-well plate. The cells were seeded in 96-well plates at 200 µL/well and cultured overnight at 37 °C for attachment. The cells were treated with CnTPPs for 17 h, then the confluency percentage was estimated by microscopy. All estimations were carried out in triplicate in three randomly selected fields of view of the microscope.

### 4.8. MTT Assay

Cell viability was evaluated by a widely used MTT, as modified in [19]. An appropriate concentration of C_12_TPP in DMEM (with 2 mM L-glutamine and 10% fetal bovine serum) was used. After being cultivated at 37 °C in 5% CO_2_/95% air for 24 h, cells were inspected by microscopy and were then washed twice with the medium, and the MTT solution was added (1 mg/mL in DMEM) for 3 h at 37 °C. The dye solution was then removed, 100 µL DMSO per well was added, and absorbance at 570/660 nm was measured. The experiments were performed twice, in which 4 wells per each concentration were analyzed.

### 4.9. Adhesion Alteration Estimation

For a few days, 293T cells were grown in flasks, treated with trypsin (0.25%), harvested, and resuspended in fresh medium (DMEM with 2 mM L-glutamine and 10% fetal bovine serum with streptomycin (100 U/mL), and penicillin (100 U/mL)). The cells were treated with C_12_TPPs (0.5 μM, 1 μM, and 5 μM) for 30 min, seeded in 96-well plates at 200 µL/well (25 μL treated cells in 175 μL fresh media), and cultured overnight at 37 °C for attachment. Cells without addition of C_12_TPP were taken as controls. Experiments were carried out in quadruplicate and qualitative estimations were carried out in selected fields of view of the microscope. The adhesion area was analyzed and measured using ImageJ software [49].

## 5. Conclusions

Although the absence of cytotoxicity is one of the most important criteria for promising antibiotics, the difficulty often lies in the fact that it is not always possible to reliably assess this. Even with high concentrations, we cannot reliably state that we are observing a cytotoxic effect, and not adhesion alteration, especially in the case of 293T cells, which can reversibly detach and reattach to the surface. Therefore, we can only state that at submicromolar concentrations of CnTPP, a decrease in metabolism begins, and the weakening of cell adhesion begins to be noticeable at concentrations above 5 μM. Only at concentrations of tens of micromoles, with an increase in the detergent effect, does the cytocidal effect also begin. This provides the necessary therapeutic window for the use of antibacterial bactericidal substances based on triphenylphosphonium derivatives; however, this should be performed with caution, keeping in mind that the border of cytocidality due to alteration of cell adhesion is very difficult to establish.

## Figures and Tables

**Table 1:** Threshold concentrations of C_12_TPP (μM) causing a reduction in luminescence and a bactericidal effect.

Effect	*E. coli*	*B. subtilis*	*V. aquamarinus*
Luminescence reduction	2.0 ± 0.6	0.25 ± 0.09	1.0 ± 0.3
Survival rate reduction	70 ± 15	1.0 ± 0.5	35 ± 17

**Table 2:** The percentage of the surface of a well that is covered by adherent.

CnTPP	GI (n)<	[C], μM	Confluence, %
C_8_TPP	50	0.2	~100
	80	2	~100
	90	4	~100
C_10_TPP	50	0.2	~100
	80	1	~100
	90	2.5	~100
C_12_TPP	50	0.05	~100
	80	0.1	~100
	90	0.5	~100

## Data Availability

Not applicable.

## Abbreviations

TPP, triphenylphosphonium; C_n_TPP, alkyltriphenylphosphonium cation; SkQ1, 10- (plastoquinonyl)decyl triphenylphosphonium; MIC, minimal inhibitory concentration; MBC, minimum bactericidal concentration; CMC, critical micelle concentration;, ECAR, extracellular acidification rate; GI, growth inhibition; MTT, 3-(4,5-dimethylthiazol-2-yl)-2,5-diphenyltetrazolium bromide; MTS, 3-(4,5-dimethylthiazol-2-yl)-5-(3-carboxymethoxyphenyl)-2-(4-sulfophenyl)-2H- tetrazolium; XTT, 2,3-bis-(2-methoxy-4-nitro-5-sulfophenyl)-2H-tetrazolium-5-carboxanilide; WST, water-soluble tetrazolium salts; ROS, reactive oxygen species; CFU, colony-forming unit.
